# Exosome mediated miR-155 delivery confers cisplatin chemoresistance in oral cancer cells via epithelial-mesenchymal transition

**DOI:** 10.18632/oncotarget.27531

**Published:** 2020-03-31

**Authors:** Prathibha Kirave, Piyush Gondaliya, Bhagyashri Kulkarni, Rakesh Rawal, Rachana Garg, Alok Jain, Kiran Kalia

**Affiliations:** ^1^Department of Biotechnology, National Institute of Pharmaceutical Education and Research, Ahmedabad, Gujarat, India; ^2^Department of Life Science, Gujarat University, Ahmedabad, Gujarat, India; ^*^These authors contributed equally to this work and are first authors

**Keywords:** cisplatin chemoresistance, miRNA155, exosomes, oral cancer, apoptosis

## Abstract

Cisplatin is used as chemotherapeutic drug for oral squamous cell carcinoma (OSCC). However, OSCC cells develop resistance following long-term cisplatin exposure. Resistance against cisplatin chemo-therapy is accredited to the process of epithelial-to-mesenchymal transition, which in-turn has been linked to tumor-recurrence. miRNA deregulation, a common event in cancer, plays contributory role in chemo-resistance. Exosomes acts as the natural cargo for miRNA and facilitates inter-cell communication in the tumor micro-environment. Hence, exosomal-mediated miRNA transference may play essential role in drug resistance and serve as a target for cancer-therapy. miR-155 upregulation in OSCC has been described, however, its relevance in the observed chemo-resistance is unclear and also, if exosomes have any role in miR-155 regulation remain elusive. In the present study, we document for the first time the critical role of exosomes in mediating increments in miR-155 expression in OSCC cells that have acquired cisplatin resistance (cis^Res^ cells). Importantly, exosomal transfer from cis^Res^ to the cisplatin sensitive (cis^Sens^) cells was found to confer significant miR-155 induction in the recipient cis^Sens^ cells. Restoration of miR-155 expression in cis^Sens^ cells following miR-155 mimics treatment led to epithelial to mesenchymal transition, enhancements in their migratory potential as well as acquisition of resistant phenotype. Notably, similar augmentations in the migratory and chemo-resistant traits were seen upon delivery of exosomes from cis^Res^ to the recipient cis^Sens^ cells. Overall, our findings establish the significance of exosomal-mediated miR-155 shuttling in the cisplatin-chemoresistance, commonly observed in OSCC cells, thereby providing rationale for targeting miR-155 signalling for oral cancer therapy.

## INTRODUCTION

Oral squamous cell carcinoma (OSCC) ranks sixth amongst all cancers worldwide, and one of the most predominant and leading cancer found in Indian sub-continent [1, 2]. Despite substantial developments in current treatment strategies, OSCC remains one of the most common cause of cancer related deaths and there are not much improvements in the 5-year survival rate over the last two decades. Recent studies have identified several contributory reason for this: lack of targeted therapy, tolerance of drug in the body, limited treatment options and a poor understanding of OSCC biology [3, 4]. There are multiple etiological factors that are responsible for oral cancer development; smoking being the primary risk factor with others including the use of alcohol, betal leaf, areca nut and, human papillomavirus (HPV) infection [5]. Though there are different options (surgery, chemotherapy and radiotherapy) available for OSCC patients, every therapy possesses advantages as well as limitations. Lethal outcomes are predominantly caused by treatment-related resistance, local recurrence and distant organ metastasis. Owing to the developing chemoresistance, significant changes occur in the tumor microenvironment of which exosomes are an important component [6].

Exosomes are extracellular vesicles (30–100 nm), that acts as cargo for mRNAs, miRNAs, DNA fragments, proteins as well as apoptotic bodies [7]. Expression pattern of exosomal-derived miRNAs differ greatly between cancer and normal control cells [8, 9], thereby hinting at the role of exosomal miRNAs as potential biomarker for cancer diagnosis, including OSCC. Several pioneering studies highlight the crucial role of exosomes in facilitating tumor growth, angiogenesis, invasion, metastasis as well as in conferring chemoresistance [10, 11]. Apart from tumor-derived exosomes, there are other factors that play important role in enhancing drug resistance, including overexpression of ABC-transporter, epithelial to mesenchymal transition (EMT), activating mutation in growth factor receptors, acquisition of cancer stem cell like properties and activation of DNA repair mechanism. Amongst these, the role of EMT in chemoresistance is of special relevance [12, 13]. Cytokines, growth factors, non-coding RNAs and hypoxia are known to elicit the EMT process, whereby epithelial cells undergo depolarization, loses their adherent property, cell-cell contact, and acquire elongated, fibroblast-like morphology. These events are associated with decreased expression of epithelial markers (E-cadherin, desmoplakin, type IV collagen, and laminin 1) and concomitant increase in mesenchymal markers (N-cadherin, integrin, vimentin, type I collagen, laminin 5, and fibronectin). The functional outcomes of EMT are majorly reflected in increased invasion, metastasis, evasion of apoptosis, drug resistance and cancer stemness [14].

Importantly, aberrant expression of small non-coding RNAs, miRNAs have been reported in multiple cancers [15, 16] and has also been shown to modulate EMT and drug chemoresistance [17–19]. For example, miR-34a has been shown to be downregulated in 5-fluorouracil (5-FU) resistant colorectal cancer, and its restoration caused regaining of 5-FU sensitivity *via* its direct target, lactate dehydrogenase A [20]. In contrast, miR-21 was found to be overexpressed in multiple cancers and its overexpression mediated cisplatin resistance particularly, in ovarian cancer *via* PTEN down-regulation [21]. Likewise, overexpression of miR-140 in colorectal cancer has been associated with chemoresistance to methotrexate and 5-FU, an effect mediated in part due to HDAC4 suppression [22]. Overexpression of miR-155 has been linked with gemcitabine resistance in pancreatic ductal adenocarcinoma [23]. It is noteworthy that miR-155 overexpression has recently been shown to function as oncomiR in oral cancer [24], it promoted proliferation, invasion and metastasis of OSCC [25]. Besides, abundance of miR-155 has been found in oral cancer patients having tobacco history compared to the non-tobacco chewers [26]. In fact, Manikandan *et al.* showed a strong association between increased miR-155 levels and the habit of chewing tobacco/betel quid in an Indian population [26]. However, effects of miR-155 on either inducing or overcoming cisplatin chemoresistance in oral cancer remains elusive. Moreover, underlying molecular mechanism (s) or the gene target (s) through which miR155 exerts its effect on cisplatin-induced chemoresistance in oral cancer remains poorly understood.

In the present study, we demonstrate for the first time that miR-155 is overexpressed in cisplatin resistant (cis^Res^) *vs* cis-senstive (cis^Sens^) oral cancer cells. In consonance to this, miR-155 upregulation was observed in oral cancer patients with disease recurrence following cisplatin treatment compared to the healthy controls or tobacco smokers with no cancer history. We further identified a transcription factor, FOXO3a as the direct target of miR-155 in cis^Res^ OSCC cells and provide evidence that miR-155 confers cisplatin resistance in OSCC cells *via* modulation of EMT pathway and downregulation of FOXO3a.

## RESULTS

### Exosome-derived miR-155 is upregulated in cisplatin resistant oral cancer

**Figure 1:**
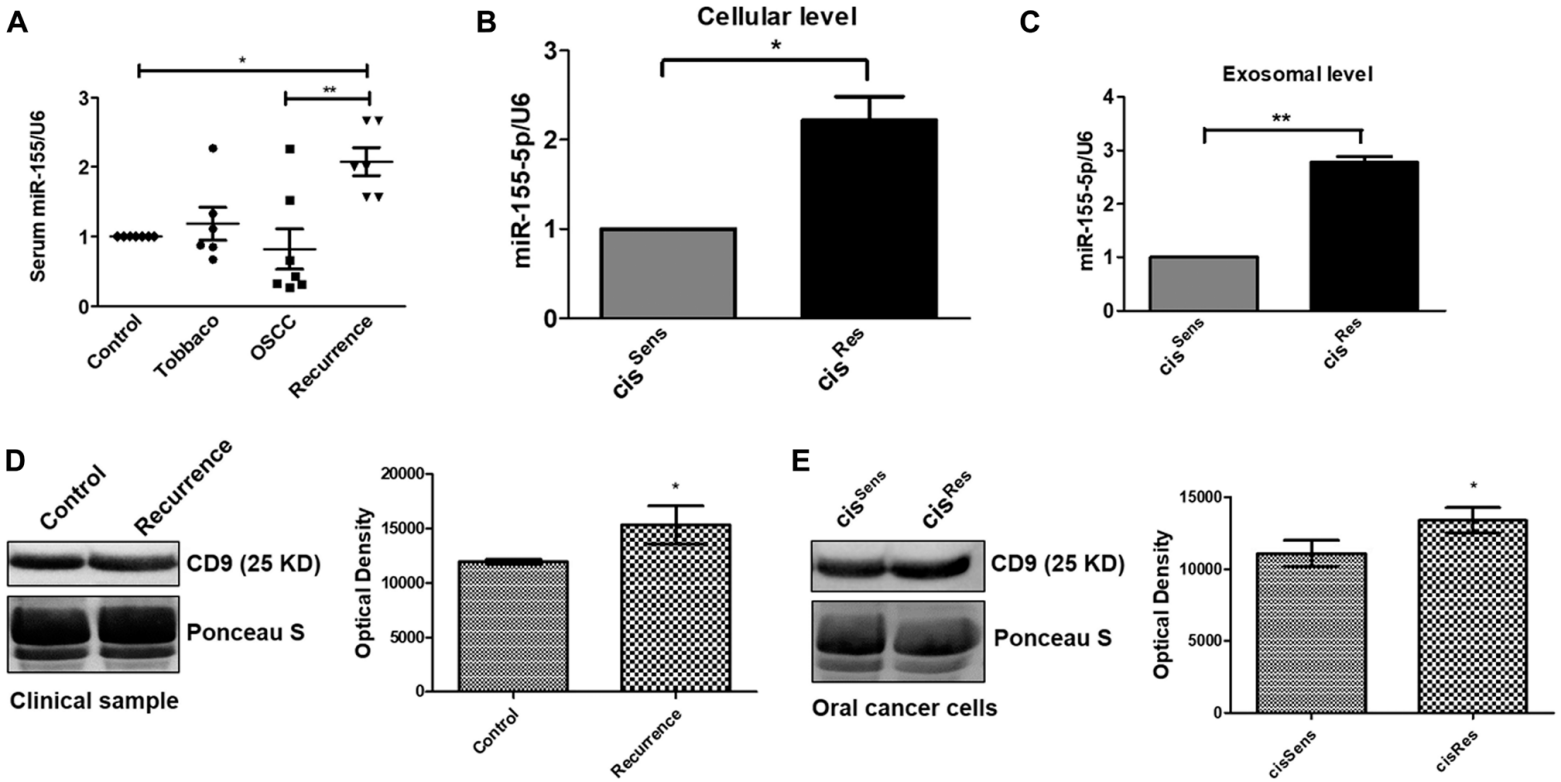
(**A**) miR-155 expression in clinical samples was quantified by q-PCR and normalized with respect to U6 as housekeeping gene. miR-155 expression profiling in cis^Res^ and cis^Sens^ oral cancer cells at the (**B**) cellular and (**C**) exosomal level. (**D** and **E**) Western blot analysis for the exosomal marker CD-9 in exosomes isolated from serum of clinical samples and cis^Res^ and cis^Sens^ SCC-131 oral cancer cells. In the absence of a well-accepted standard internal control, densitometric analysis was done by normalizing the CD9 blot with a prominent band visualized on the PVDF membrane following Ponceau-S staining. Data are expressed as mean +/– SD.^*^ *p* < 0.05 and ^**^ *p* < 0.01 compared with the cis^Sens^ cells or healthy controls in case of clinical samples. (*n* = 3). Two independent experiments gave similar results.

miR-155 is upregulated in oral cancer, however its implications in observed chemoresistance remains unclear. Hence, we first evaluated the miR-155 expression in the exosomes isolated from the serum samples of healthy control, healthy control with tobacco history but no cancer, oral cancer patients and oral cancer patients with recurrence post-cisplatin chemotherapy. Interestingly, healthy controls with tobacco history showed upregulation of exosomal miR-155 when compared to the normal healthy control volunteers (Figure 1A). Dysregulated expression of exosomal miR-155 was found in oral cancer patients, with some having high while others showed lower expression. This could be attributed to the genetic variation and mixed population under study. Notably, miR-155 isolated from exosomes of OSCC patients having disease recurrence post-cisplatin chemotherapy showed significant enhancement when compared to not only the healthy control but also to the oral cancer patients, thereby indicating at the relevance or association of miR-155 with disease recurrence and development of drug resistance (Figure 1A). These results were further corroborated *in vitro* in the OSCC cells. Remarkably, as observed in the clinical samples, miR-155 was found to be significantly overexpressed in cis^Res^ cells compared to cis^Sens^ OSCC cells. Furthermore, analysis of miR-155 expression in the exosomes isolated from cis^Res^ cells also showed significant upregulation in contrast to the exosomal miRNA for cis^Sens^ cells (Figure 1B–1C). It is important to mention here that serum from oral cancer patients having disease recurrence showed higher amount of exosomal content than that in normal healthy controls, as analysed by the expression of exosomal marker CD9, indirectly accounting for the increased miR-155 expression in recurrent tumors (Figure 1D). Exosomal increment was also seen in the cis^Res^ cells compared to cis^Sens^ cells (Figure 1E). Overall, results indicates that exosomal-mediated miR-155 upregulation in oral cancer is associated with cisplatin resistance.

### *FOXO3a* is the gene target of miR-155 in oral cancer cells

**Figure 2:**
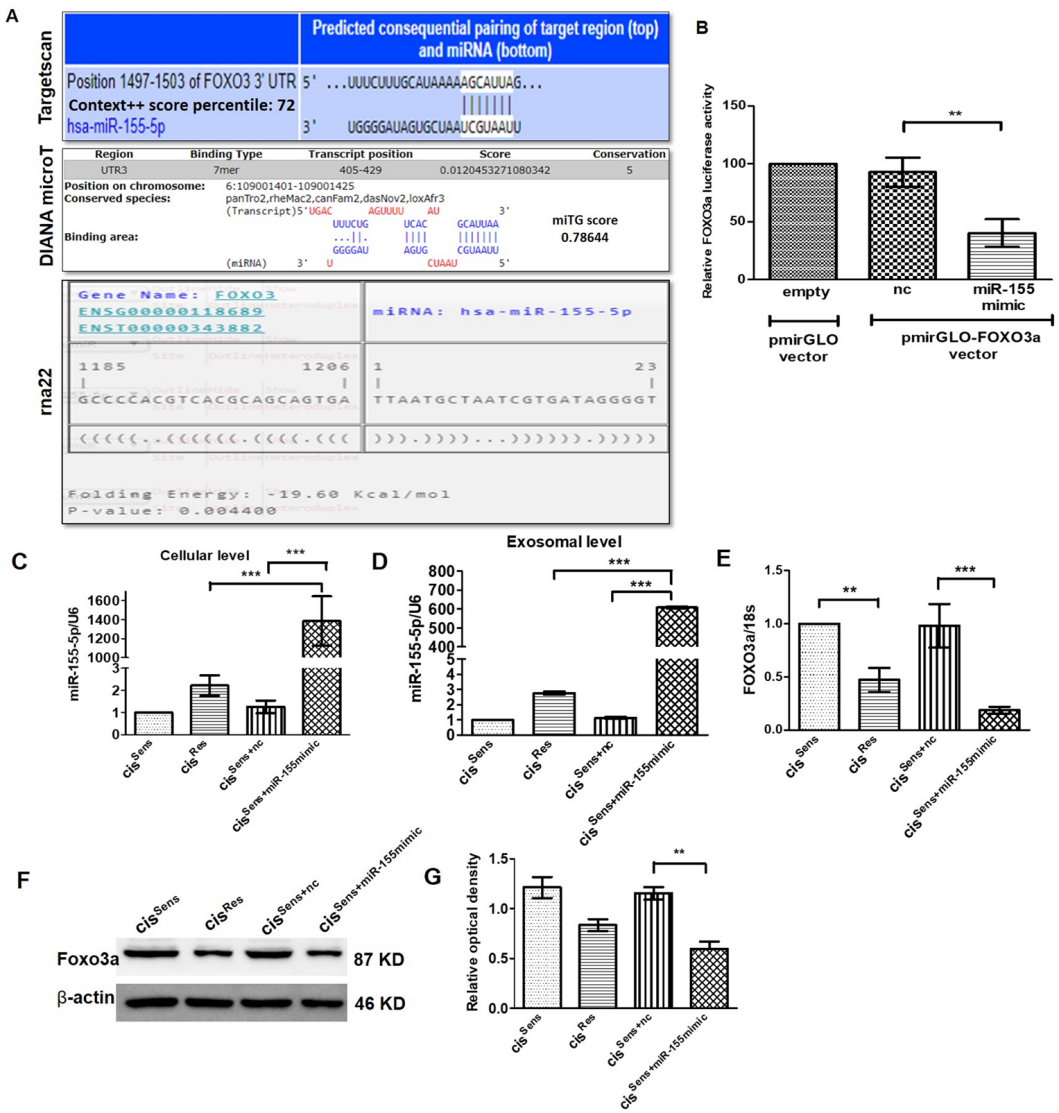
(**A**) Binding position prediction of miR-155 with FOXO3 using TargetScan, DIANA microT-CDS and rna22 web-based tools. (**B**) FOXO3a luciferase activity in cis^Sens^ cells co-transfected with either NTC or miR-155 mimics and the cloned p-mirGLO-FOXO3a dual luciferase vector. Data are expressed as the mean +/– SD. ^**^ *p* < 0.01, significant difference vs. NTC group (*n* = 3). Two independent experiments gave similar results. Following transfection of miR-155 mimics in cis^Sens^ cells, miR-155 expression was validated by q-PCR at both the (**C**) cellular and (**D**) exosomal level. FOXO3a expression was measured by (**E**) q-PCR and (**F**) Western Blot. (**G**) Densitometry analysis of FOXO3a western blot normalized to β-actin as the loading control. Data are expressed as the mean +/– SD. ^*^ *p* < 0.05 and ^**^ *p* < 0.01. (*n* = 3). Two independent experiments gave similar results.

miRNAs are known to regulate gene expression by binding to the 3′-untranslated region (UTR) of their targets mRNA; thereby curbing mRNA stability and diminishing protein translation. In the present study, we intended to identify the gene targets of miR-155 in oral cancer and establishing their role in chemoresistance. Towards this end, we have used three different web-based tool, each of which employed different algorithms to predict the gene-targets (discussed in the method section). Targetscan [27], DIANA micro-T [28] and RNA22 [29] predicted a total of 556, 1084 and 4639 gene targets respectively for miR-155. Out of these, the top 100 gene targets were considered for further screening based on their Context++ score percentile (Targetscan), miTG (DIANA micro-T) and folding energy (RNA22) score. Furthermore, only those gene targets that are known to be associated with drug resistance were short-listed. This screening thus generated a new table having 18, 18 and 5 gene targets that were originally identified by Targetscan, DIANA micro-T and RNA22 respectively (Supplementary Table 1). Finally, following a consensus approach, we identified *FOXO3a* as miR-155 target. It is one of the three genes (FOXO3, DYNC1/1 and CARNSP1) that were positively predicted by all the three web-based tools used (Figure 2A and Supplementary Table 3). Interestingly, amongst them, only *FOXO3a* was found to have association with oral cancer [30].

Following *in-silico* prediction, we validated that *FOXO3a* acts as miR-155 gene target using a dual luciferase reporter assay approach. For this analysis, we co-transfected cis^Sens^ cells with either *NTC* or miR-155 mimics and the cloned p-mirGLO-FOXO3a dual luciferase vector. As seen in Figure 2B, comparable FOXO3a luciferase activity was observed in cells transfected with *NTC* and empty vector. Remarkably, FOXO3a luciferase activity was significantly reduced in the cis^Sens^ OSCC cells upon miR-155 mimics transfection, indicating that miR-155-mediates regulation of FOXO3a. We verified the transfection efficiency of miR-155 mimics by qPCR. As mentioned before (Figure 1B and 1C), cis^Res^ cells showed a significant abundance of cellular as well as exosomal miR-155 levels compared to the cis^Sens^ cells (Figure 2C and 2D). Notably, miR-155 mimics transfection in cis^Sens^ resulted in marked upregulation of miR-155 expression both at the cellular and exosomal level in contrast to the cells transfected with *NTC* (cis^Sens-NTC^). Notably, cis^Res^ cells, wherein elevated miR-155 levels were observed showed significant decrease in the *FOXO3a* mRNA and protein expression when compared to the cis^Sens^ cells (Figure 2E–2G). It is important to note that, miR-155 mimics transfection in cis^Sens^ (cis^Sens+miR-155mimic^) cells led to a significant reduction in both mRNA and protein levels when compared to cis^Sens+NTC^ or even cis^Res^ cells. Results thus clearly indicate that miR-155-mediates downregulation of its target gene, *FOXO3a* in OSCC cells and this effect is more pronounced in the cis^Sens^ cells following the miR-155 restoration.

### miR-155 upregulation mediates epithelial to mesenchymal transition in cis^Res^ OSCC cells

**Figure 3:**
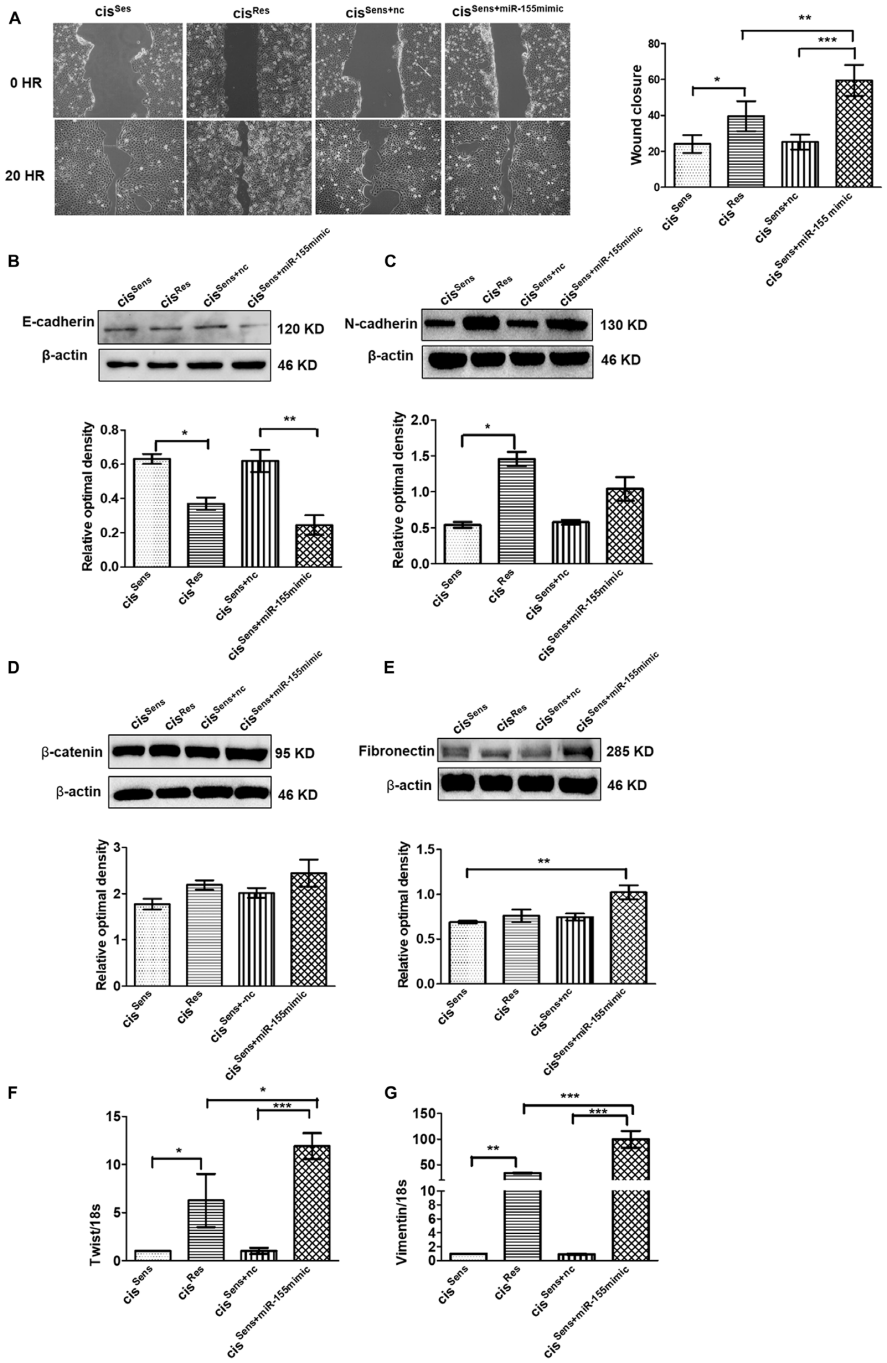
(**A**) Effects of cisplatin treatment was analyzed on the migration of cis^Sens^ oral cancer cells transfected with either miR-155 mimics or NTC (non-target control) by wound assay. cis^Res^ cells were employed for the comparison. Left panel, Representative images of wound closure taken at 0 and 24 h after the scratch was made and cisplatin treatment initiated. Right panel, quantification of wound closure as analyzed using Image J. Western Blot expression of various EMT associated markers was measured. Subsequently densitometry analysis was done normalized to β-actin as the loading control. The protein markers included: (**B**) E-cadherin, (**C**) N-cadherin, (**D**) β-catenin, and (**E**) Fibronectin. (**F**) Twist and (**G**) Vimentin expressions were quantified by q-PCR and normalized with respect to 18S as the housekeeping gene. Data are expressed as mean ± SD. ^*^ *p* < 0.05 and ^**^ *p* < 0.01, ^***^ *p* < 0.001. (*n* = 3). Two independent experiments gave similar results.

Emerging evidences have linked miR-155 with migration and metastasis in multiple cancers [25, 31], however its relevance in oral cancer with drug resistance is not clear. We therefore next intended to evaluate the effect of miR-155 on the migration of cis^Res^ cells by scratch assay. Notably, cisplatin resistance in OSCC mediated enhanced migration as can be seen in the extent of wound closure in the cis^Res^ *vs* cis^Sens^ OSCC cells (Figure 3A). Furthermore, restoration of miR-155 in cis^Sens^ cells using mimics approach accelerated the wound closure in comparison to the parental cis^Sens^ or cis^Sens+nc.^ Importantly, the migration rate in cis^Sens^ cells upon miR-155 restoration was comparable to that observed in cis^Res^ cells. This is in agreement with the enhanced cisplatin resistance observed in cis^Sens^ cell following miR-155 mimic transfection (data not shown).

Having observed the essential role of miR-155 on cell migration with respect to cisplatin resistance, we next evaluated its effect on the markers related to EMT pathway. As can be seen in Figure 3B–3G, cis^Res^ OSCC cells (that possess elevated miR-155 levels) exhibited mesenchymal properties, which is a characteristic of advanced and aggressive cancerous condition. In concordance to this, cis^Res^ cells showed elevated levels of EMT markers such as N-cadherin, twist and vimentin with concomitant reduction in E-cadherin levels. To conclusively establish if mesenchymal phenotype in cis^Res^ OSCC cells is driven by miR-155, we next evaluated the expression of various EMT markers after restoring miR-155 levels in the cis^Sen^ OSCC cells. Of note, Cis^Sens^ miR-155 cells showed a significant reduction in E-cadherin expression with a concomitant increase in the expression of N-cadherin, vimentin, and twist (Figure 3B–3G). Expression of the mesenchymal markers, β-catenin and fibronectin showed only marginal increase in the cis^Res^ or cis^Sens+miR-155mimic^. These results therefore suggest that miR-155 overexpression facilitates the expression of genes associated with the maintenance of the mesenchymal phenotype in the cis^Res^ OSCC cells; as is also reflected in the cis^Sens^ cells following miR-155 restoration.

### Essential role of exosomal miR-155 in conferring cisplatin resistance and cell growth

**Figure 4:**
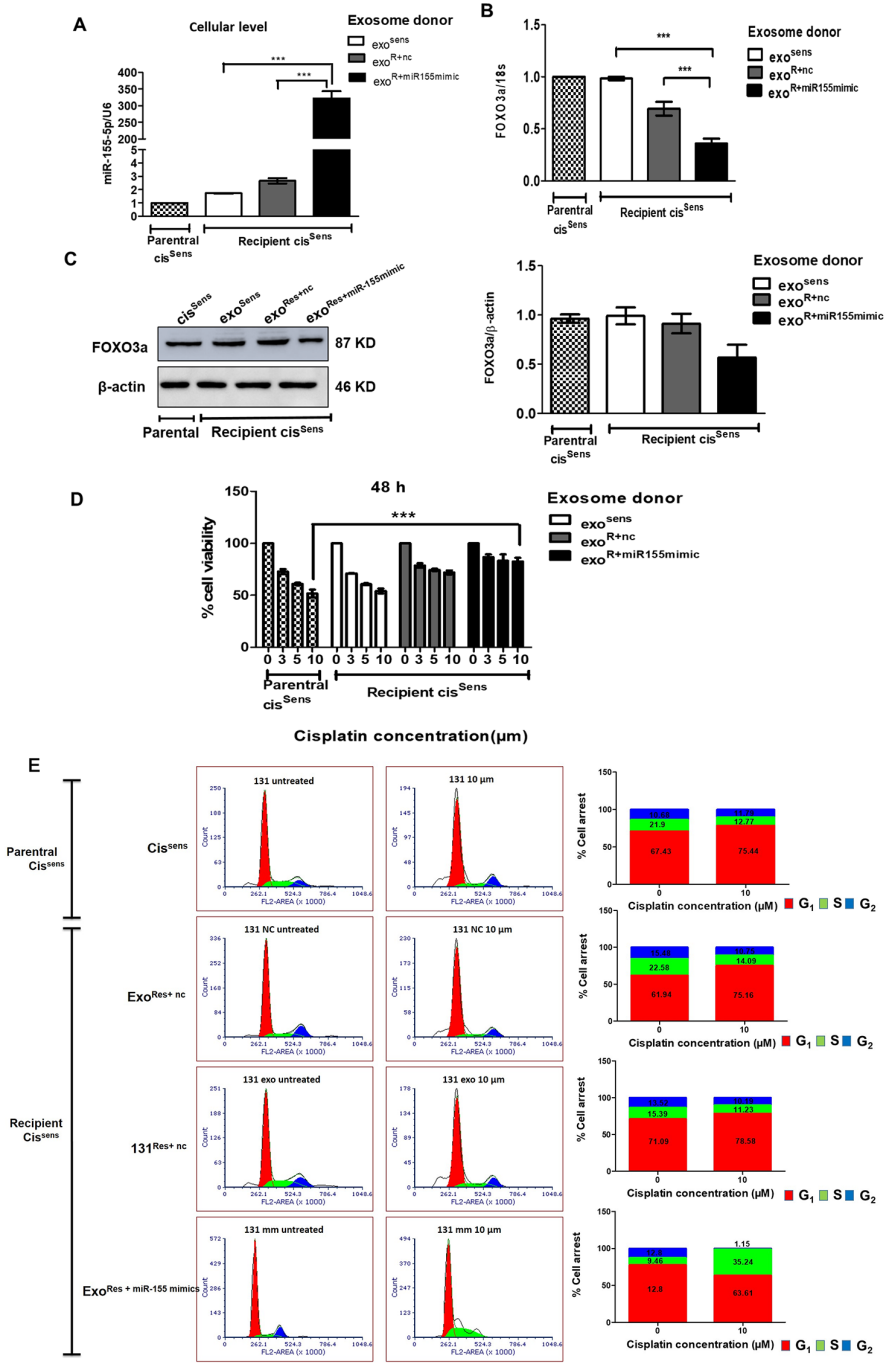
Exosomes were isolated from cis^Sens^ and cis^Res^ cells transfected with either *NTC* or miR-155 mimics, and were used to treat naïve cis^Sens^ cells. cis^Sens^ cells were employed as controls and for comparison. (**A**) miR-155 were quantified by q-PCR and normalized with respect to U6 as housekeeping gene and (**B**) FOXO3a expression was quantified by q-PCR and normalized with respect to 18S as housekeeping gene. (**C**) FOXO3a expression was measured by Western Blot. Densitometry analysis of FOXO3a western blot normalized to β-actin as the loading control. (**D**) Following exosomes conditioning treatment, cells were exposed to different concentrations of cisplatin (μM, as indicated) for 48 h. Cell viability was measured by the MTT assay. Data are expressed as the mean +/– SD. ^*^ *p* < 0.05 and ^**^ *p* < 0.01 (*n* = 3). Two independent experiments gave similar results. (**E**) After cisplatin treatment, cells were harvested, stained with PI and analysed for cell distribution into different phases of cell cycle using FACS S3e cell sorter (Bio-Rad).

Tumor microenvironment greatly influences the migratory behaviour of cancer cells and their acquisition of drug resistance. Exosomes, being the key component of tumor microenvironment, we next evaluated if they facilitate communication between cis^Sens^ and cis^Res^ cells and hence their resistant or migratory potential. For this, we adopted a co-treatment approach: first, we isolated exosomes from three different combinations of cells: cis^Sens^, cis^Res^ cells transfected with either miR-155 mimics (cis^Res+miR-155mimic^ cells) or *NTC* (cis^Res+nc^ cells), and thereafter used the isolated exosomes to treat naïve cis^Sens^ cells. As seen in Figure 4A, incorporation of exosomes from cis^Res+miR-155mimic^ into naïve cis^Sens^ cells led to significant enrichments in their miR-155 expression, while a marginal increase in miR-155 expression was seen when the exosomal donor was cis^Res+nc^ cells. No obvious difference was noticed in case of exosomal transfer from cis^sens^. Overall results indicate that miR-155 mimics-mediated increase in cellular miR-155 expression led to enrichment of exosomal miR-155 content (Figure 2C–2D) and their transfer in cis^Sen^ cells, in turn resulted in augmentation of miR-155 abundance in cis^Sen^ cells (Figure 4A). Furthermore, in corroboration to miR-155 endowment, significant downregulation in both the protein and mRNA levels of its target, *FOXO3a* was observed in the cis^Sens^ cells receiving exosomes from cis^Res+miR-155mimic^ cells (Figure 4B and 4C). Of note, exosomal-mediated delivery of miR-155 in cis^Sens^ OSCC cells enhanced their resistance towards cisplatin. The effect is more pronounced when exosomal donor cells is cis^Res-miR-155mimic^ and cisplatin dose is 10 μm (Figure 4D). Besides, exosomes mediated enhanced transfer of miR-155 resulted in increased cell numbers in G0/G1 phase with a decrease in G2/M phase, indicating a removal of cell cycle arrest, as otherwise noted in cis^Sens^ or cis^Res+nc^ (Figure 4E). Results thus highlight the role of exosomal miR-155 trafficking in transferring chemoresistance by abrogating the cisplatin induced cell cycle arrest in cis^Sens^ cells.

### Exosomal mediated miR-155 delivery enhanced migration and mesenchymal traits in cis^Sens^ cells

**Figure 5:**
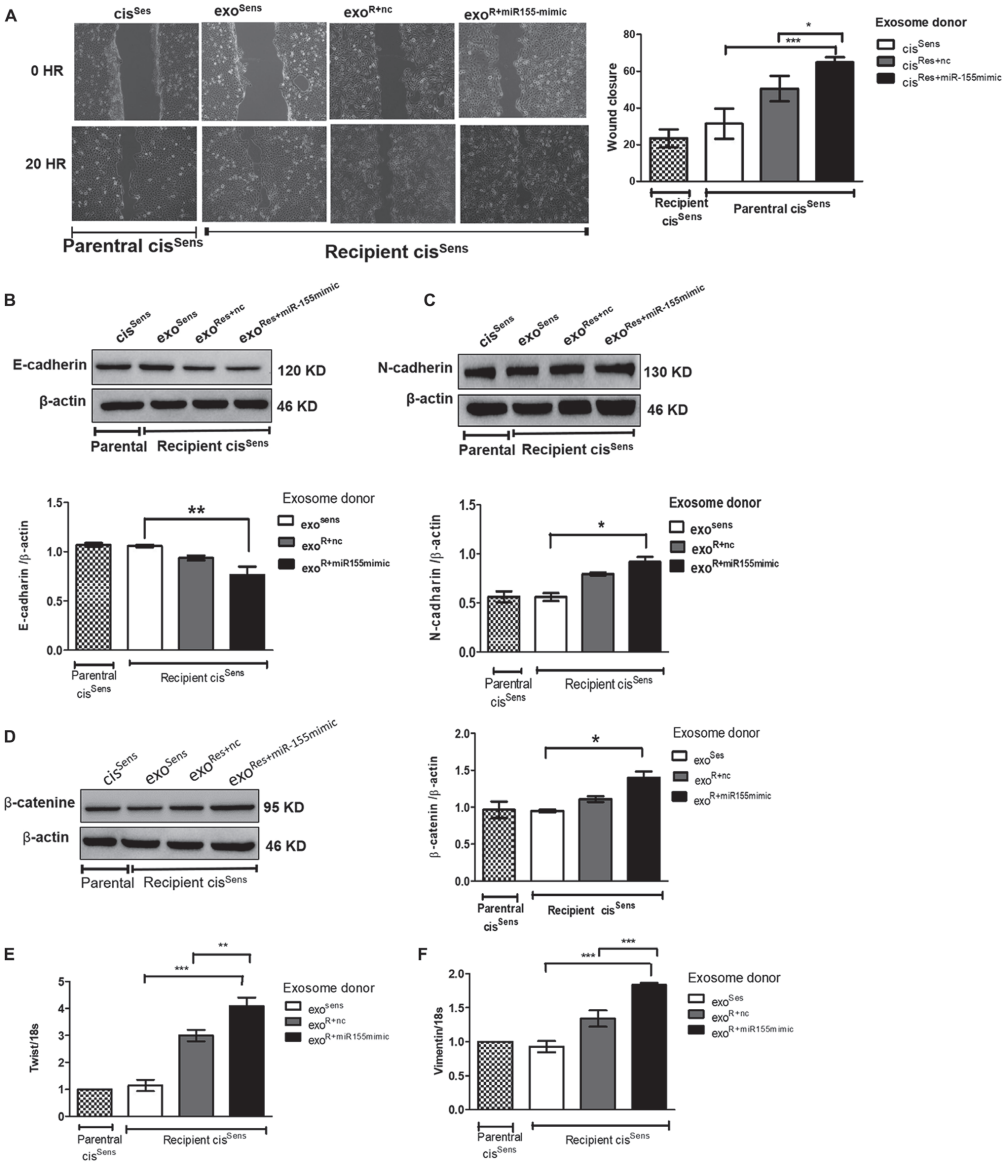
Exosomes were isolated from cis^Sens^ and cis^Res^ cells transfected with either *NTC* or miR-155 mimics, and were used to treat naïve cis^Sens^ cells. cis^Sens^ cells were employed as controls and for comparison. (**A**) Effects of cisplatin treatment was analyzed on the migration of cis^Sens^ oral cancer cells receiving exosomes from cis ^Res+miR-155 mimic^ cells by wound assay. *Left panel*, Representative images of wound closure taken at 0 and 24 h after the scratch was made and cisplatin treatment initiated. *Right panel*, quantification of wound closure as analyzed using Image J. Western Blot expression of various EMT associated markers was measured. Densitometry analysis was carried out with β-actin as loading control. The protein markers included: (**B**) E-cadherin, (**C**) N-cadherin, and (**D**) β-catenin, (**E**) Twist and (**F**) Vimentin expressions were quantified by q-PCR and normalized with respect to 18S as the housekeeping gene. Data are expressed as mean ± SD. ^*^ *p* < 0.05 and ^**^ *p* < 0.01, ^***^ *p* < 0.001 (*n* = 3). Two independent experiments gave similar results.

Having observed the effects of exosomes on enhancing miR-155 abundance and transmitting chemoresistance in cis^Sens^ cells, we next intended to determine if exosomal-mediated crosstalk has any influence on their migratory potential as well. Remarkably, exosomal shuttling of miR-155 from cis^Res+miR-155mimic^ to parental cis^Sens^ cells led to the faster closure of wound (i. e. enhanced cell motility) compared to the condition wherein cis^Res+nc^ or cis^Sens^ acts as the exosomal donor (Figure 5A). EMT is known to play key role in tumorigenesis and metastatic events in various cancer types, via modulating cell motility and invasiveness. Indeed, in the present study, effects of exosomal miR-155 transfer were also reflected in conferring mesenchymal properties to the parental cis^Sens^ cells (Figure 5B–5F). As seen in the figure, there are significant enhancements in the expression of mesenchymal markers including N-cadherin, β-catenin, twist and vimentin with the concomitant decrease in epithelial marker, E-cadherin in cis^Sens^ cells conditioned with exosomes from cis^Res+miR-155mimic-^ cells. Significant effects were also noticed in the expression of mesenchymal markers (vimentin and twist) in cis^Sens^ cell receiving exosomes from cis^Res+nc^, thus hinting that exosome are sufficient to elicit the EMT response. cis^Sens^ cells receiving exosomes from cis^Sens^ itself showed comparable expression with the parental cis^Sens^. Overall, results suggest that exosomal-mediated transmission of miR-155 leads to enhanced cell motility and mesenchymal transition.

## DISCUSSION

Chemoresistance, being a major cause of tumor recurrence, is one of the major hurdle in oral cancer treatment and management. Several factors including EMT, drug efflux, DNA damage repair, cell-death inhibition, drug-target alteration, drug-inactivation, epigenetics are some of the chief causes that contribute to chemoresistance [32]. Pioneering studies have highlighted the key role of growth factors, cytokines, transcription factors, non-coding RNAs, and tumor microenvironment in triggering EMT and acquisition of drug resistance [3]. Exosomes (mediators of cell-to-cell communication) transfer molecular and genetic information’s from tumor to both normal and abnormal cells present in the tumor microenvironment [33]. In the present study, we present evidence showing the critical role of exosomal miR-155 in transferring cisplatin resistance, which in turn evaded cell cycle arrest, enhanced cell motility and conferred mesenchymal traits in the cisplatin sensitive OSCC cells.

Role of miRNA in drug resistance has recently been appreciated and documented in various studies [34, 35]. For instance, overexpression of miR-21 has been shown to mediate cisplatin resistance in ovarian cancer *via* negatively regulating PTEN, a tumor suppressor protein [36]. Likewise, upregulation of miR-140 in colon cancer stem-like cells is accredited to 5-FU resistance [22]. In a study by *Santos et al*. exosomal-mediated transfer of miR-155 was shown to cause doxorubicin as well as paclitaxel resistance in breast cancer [37]. Herein, we found the upregulation of miR-155 in healthy controls but with tobacco history when compared to controls, this is in concordance with the earlier studies conducted in tobacco/betel-quid chewers and cigarette smokers [38]. The importance of our study is heightened by the fact that we present here the first evidence demonstrating significant enhancements in miR-155 expression in the oral cancer patients specifically with disease recurrence post-cisplatin treatment as compared to not only the healthy controls but also the oral cancer patients. Furthermore in the present study, abundance of both cellular and exosomal miR-155 was observed in cis^Res^ *vs* cis^Sens^ oral cancer cells thus indicating at the essential role of miR155 in cisplatin resistance. A recent miRNA profiling study however, showed miR-155 down-regulation in cis^Res^ OSCC cells [39]. It is important to consider that in this study, authors have conducted miRNA profiling solely based on existing databases, and had validated only a few miRNA, which did not included miR-155 [39].

Exosomes contribute to cancer progression as well as chemoresistance in various cancer types *via* modulating immunosuppression, angiogenesis, invasion and metastasis [6]. They act as cargos for proteins, nucleic acids (mRNAs, non-coding RNAs, DNA sequences) as well as lipid molecules thereby playing essential roles in intercellular communication [40]. To this end, *Valadi et al.* [41] showed that the RNA from mast cell exosomes were transferable to other mouse and/or human mast cell where they were fully functional and were capable in modulating targeted protein production. In agreement with the messengerial role of exosomes, our study documents for the first time the relevance of exosomal-mediated miRNA (miR-155) transfer in conferring drug resistance from cis^Res^ to cis^Sens^ OSCC cells and hence its implication in the disease recurrence.

Dysregulated miRNAs expression has been linked with cancer progression, metastasis and chemoresistance. miR-760, for example was found to be downregulated in breast cancer cells, whilst its forced overexpression caused the sensitization towards doxorubicin *via* EMT reversal [42]. Likewise, miR-3129 upregulation mediated suppression of bufalin chemoresistance in epithelial ovarian cancer *via* CD44 regulation [43]. In contrast, upregulation of miR-30a, miR-375 and miR-376 in melanoma, cervical and ovarian cancer mediated resistance to chemotherapeutic drugs: cisplatin and paclitaxel [44–46]. Exosomal-mediated delivery of miR-155 has been shown to transfer paclitaxel and doxorubicin resistance in breast cancer cells [37]. A miRNA profiling study using PhenomiR database v2.0 showed miR-155 downregulation in oral cancer cells, however the *in silico* results have never been validated for miR-155. In contrasts to this, in the present study, we report the exosomal overexpression of miR-155 in oral cancer patients with disease recurrence post-cisplatin treatment when compared to cancer patients or healthy controls. miR-155 upregulation was also correlated with the tobacco exposure in the otherwise healthy individuals. Furthermore, we showed that miR-155 is upregulated both at the cellular as well as exosomal levels in the OSCC cell lines that were cisplatin resistant. Of note, the strength of our study is further enhanced in demonstrating the exosomal miR-155 delivery from cis^res^ to cis^Sens^ OSCC cells not only enhanced their miR-155 abundance but also transfered the cisplatin resistance trait in conjuction with the release of cell cycle arrest, increased motility and mesenchymal phenotype. Indeed, EMT has been shown as a root of chemoresistance [47] however, if it is related to dysregulated miR-155 expression in oral cancer remained unexplored. Besides, in the present study, we identified a transcription factor, FOXO3a as a direct target of miR-155 and showed that its negative regulation is associated with cisplatin resistance in oral cancer. Notably, a recent study in glioma cells also ascertained the role of miR-155 in regulating cell proliferation and invasion *via* FOXO3a [48], however its association with drug-resistance was not evaluated. In agreement with our findings, *Lu et al.* have shown that FOXO3a overexpression inverses platinum resistance in ovarian cancer [49].

**Figure 6:**
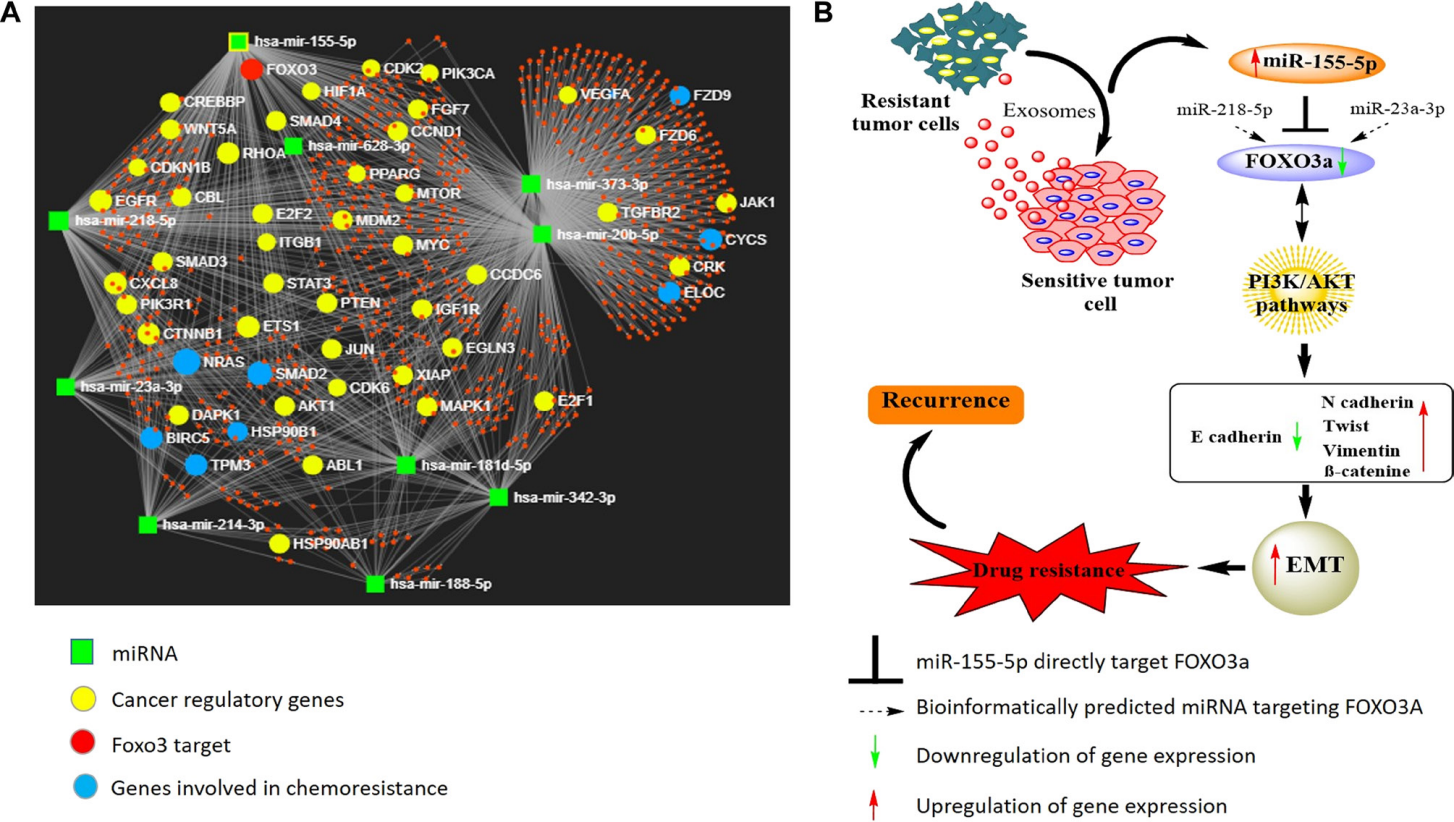
(**A**) Diagram illustrating the network pattern generated by miRNet analysis. The miRNet webserver (http://www.mirnet.ca) was used to create the network. (**B**) Predicted model illustrating the role of exosome-mediated miR155-5p transference and effects on its target gene in conferring cisplatin resistance in OSCC cells.

miRNAs are known to subtly affect the expression of targeted genes and trigger modest phenotypic effects. In addition, some miRNAs regulate single gene synergistically [50]. It will therefore be interesting as well as informative to evaluate both the collective functions of the target genes regulated by multiple miRNAs, as well as cooperation of two or more miRNAs affecting the expression of same target gene. To this end, we conducted a thorough literature search and identified ten miRNAs (including miR-155) that have been associated with cisplatin resistance in oral cancer [51]. Thereafter, we employed miRNet network visual analytics system [52] (http://www.mirnet.ca) to generate a network pattern (based on 11 publicly available databases) and evaluated the base paring between the identified 10 miRNAs and their targets (Figure 6). The generated network also provided us the list of genes associated with cancer progression. Notably, besides miR-155 through this network analysis we identified two other miRNAs: miR-23a-3p and miR-218-5p 5 that may also regulate FOXO3a expression. It will be interesting to study the synergistic effects of these three miRNA on the regulation of FOXO3a expression, however, it is beyond the scope of the present manuscript and will be our next target of study.

Taken together, our results provide mechanism of transmission of cisplatin-resistance in oral cancer *via* exosomal miR-155 and identifies the relevance of its target FOXO3a in mediating chemoresistance. Thus, miR-155 targeting therapies when combined with conventional chemotherapy might be helpful to combat chemoresistance.

## MATERIALS AND METHODS

### miR-155 target prediction

Several bioinformatics tools are reported to predict miRNA targets [53]. To overcome any ambiguity in the prediction of miR-155 target gene, we have employed three different tools namely: Targetscan [27], DIANA micro-T [28] and RNA22 [29]. Targetscan web-based tool predicts based on the following features: seed match, 3′ complementarity, local adenine uracil content and position contribution. DIANA micro-T CDS on the other hand considers free energy of binding and complementarity to predict the target gene. RNA22 assesses the possible targets by analysing their recognising pattern as well as folding energy. Finally, based on the consensus approach, target gene is chosen.

### Patient samples

OSCC patients’ blood samples were obtained from Dr. Rakesh Rawal, Department of Life Science, Gujarat University who has the approval for the study from the Institutional Ethical Committee of Gujarat University (No. GU/IEC/10/2018). Samples were processed as per the described protocol to obtain serum [54]. OSCC patients samples were categorised into four groups: healthy volunteers (*n* = 7), healthy smoker/tobacco (*n* = 6) primary tumor (*n* = 7), and recurrence tumor patient that had received cisplatin treatment (*n* = 6).

### Cell culture

Human oral squamous cell carcinoma cell lines SCC131 (cis^Sen^) was received from Dr. Susanne Gollin (Department of Human Genetics, University of Pittsburgh, USA) [55] and its cisplatin-resistant derivative (cis^Res^) from Dr. Ruma Dey Ghosh (Tata Translational Cancer Research Centre, Kolkata, India). Cells were cultured in Dulbecco’s modified Eagle’s medium with 10% fetal bovine serum, 1% penicillin-streptomycin at 37°C. cis^Res^ cells were treated with cisplatin (Sigma-Aldrich) at a concentration of 1 μg/ml every alternate passage [39], however, during experimental duration, cells were grown without cisplatin.

### Exosome isolation

Exosome isolation was performed as described with some modifications [56]. 200 μl serum and 5 ml cell culture medium were used to isolate exosomes from oral cancer patients and OSCC cells respectively. Cell debris and apoptotic bodies were eliminated using a series of differential centrifugation carried out at 4°C: first 300 g for 10 min, then 2000 g for 15 min, and finally at 10,000 g for 30 min. To obtain the exosomal pellet, supernatant was collected and subjected to ultracentrifugation at 1,00,000 g, 4°C for 70 min. PBS was added and an additional round of ultracentrifugation was done to remove microvesicles contamination. Cleaned and purified exosomal pellets was resuspended in PBS and following protein estimation, they were stored at –80°C until further use. Exosomal preparation were confirmed by the western blot analysis of the exosomal specific surface marker, CD-9.

### Isolation of mRNA and miRNA and real time quantitative PCR (qPCR)

Total RNA from the cells was isolated using Qiazol lysis reagent (Qiagen) whereas miRNA from cultured cells and exosomes were isolated using miRNAeasy kit as per the manufacture instructions. Quantitation of both cellular RNA and miRNA were done using 2100 Bioanalyzer (Agilent Technologies). 1 μg RNA from each sample was reverse transcribed to obtain cDNA using BioRad iScript kit. Real-time PCR was then performed in triplicate with each cDNA sample (1:10 dilution) using SYBR^®^ Green master mix (BioRad), forward and reverse SYBR-green primers for FOXO3a, Twist and Vimentin (primer sequences listed in Supplementary Table 2). PCR amplifications were performed using an Applied Biosystems detection system. Taqman probe was used for the miR-155 expression analysis. For the normalization of miRNA expression, U6snRNA was used as an endogenous control, and for each of the target genes, β-actin was used as a refrence. Results were analysed using the 2^–∆∆Ct^ method.

### miRNA mimics transfection

0.5 × 10^6^ cells in 6-well plate were transfected with either miR-155 mimics or non-target control (*NTC*) using Lipofectamine RNAimax (Invitrogen) in Opti-MEM as described before [57]. After 48 h of transfection, cells were used in the respective experiments. miR155 transfection efficiency was confirmed by qPCR.

### Western blot

Western blot was performed essentially as described previously [58]. Following primary antibodies were used: CD-9 (1:2000 Abcam), FOXO-3a (1:250 Santacruz), E-cadherin (1:250 Abcam), β-catenin (1:4000, Abcam), fibronectin (1:250 Abcam) and β-actin (1:5000, Santacruz). Goat anti-mouse IgG-HRP (1:20,000, Santacruz) and goat anti-rabbit IgG-HRP (1:20000, Abcam) were used as secondary antibodies. Expression of proteins were observed using ECL substrate (BioRad) and images were captured using a ChemiDoc™ imaging system (BioRad). Densitometric analysis was carried out and the band density was normalized to β-actin as the internal control. For the exosomal characterization using CD9 as the marker, owing to the absence of a valid internal reference control for exosomes, a distinct band following Ponceau-S staining of the PVDF membrane was used as the reference for normalization, as we had done previously with microsomes [59].

### Cloning, transfection and luciferase reporter assay for miR-155-5p target gene, *FOXO3a*

3′UTR sequence of miR-155-5p target gene, *FOXO3a* and mismatch sequence were synthesized using Integrated DNA Technologies, USA (sequence detailed in Supplementary Table 3). Oligonucleotide sequences were annealed, ligated and cloned into the XbaI and PmeI restriction endonuclease sites of the pmirGLO dual luciferase vector as per the manufacturer instructions (Promega). Cloned vector was also confirmed by sequencing. 0.5 × 10^5^ cells in the 24 well plate were transfected with this pmirGLO *FOXO3a* dual luciferase vector using Lipofectamine 2000 (Invitrogen, Grand Island, NY, USA). 48 h later, cells were lysed with passive lysis buffer and Dual-Luciferase Assay system (Promega, Madison, WI, USA) was used to measure the luciferase activity on a multimode luminometer reader (Perkin Elmer, USA). Data were expressed as the ratio between *firefly* and *renilla* luciferase activities [60].

### Migration assay

Migration (wound) assay was performed using confluent mono-layer of cells transfected with either miR155 mimics or *NTC* in 6-well plate. A scratch was made using 20 μl sterile pipette tip, wound created was gently washed with PBS and finally serum free media was added. Images were taken at different time interval (0 and 20 h) using inverted microscope.

### Cell viability assay

Cell viability after cisplatin treatment was studied by MTT assay as previously described [61]. Briefly, 0.5 × 10^4^ cells/well in 96-well plate were treated with different concentrations of cisplatin (0, 3, 5 and 10 um). After 24 h of cisplatin treatment, cells were incubated for additional 4 h with 3-(4,5-dimethylthiazol-2-yl)-2,5-diphenyltetrazolium bromide (MTT solution), and subsequently DMSO was added to dissolve the violet formazan crystals. Absorbance was measured at 540 nm in the microplate reader (Thermo Scientific MultiSkan™ GO, USA). Cell viability was expressed as percent compared to control group *i. e.* without cisplatin treatment.

### Cell cycle analysis

0.5 × 10^5^ cells in 6-well plate were treated with different concentrations of cisplatin (0, 3 and 10 um) for 48 h. Cells were harvested, washed with cold PBS and fixed using 70% chilled ethanol. Cells were subsequently washed with PBS and treated with 100 μg/ml RNase A solution in PBS for 30 min at room temperature. Treated cells were further incubated with 50 μg/ml PI solution (Sigma-Aldrich) for 30 min in dark, and finally analysed for cell distribution into different phases of cell cycle using FACS S3e cell sorter (Bio-Rad).

### Exosome co-conditioning assay

Exosomes were isolated from the three different group of cells: a) Cis^Sen^, b) cis^Res^ transfected with miR-155 mimics and c) cis^Res^ transfected with *NTC* scrambled sequence. Respective exosomes (20 μg) were used to treat cis^Sen^ cells (0.5 ×10^6^ in 6-well plate) that have previously been serum-starved. After 48 h of exosomal conditioning, recipient cis^Sen^ cells were assayed for protein, RNA and miRNA analysis.

### Statistical analysis

Statistical analysis was done with GraphPad Prism Version 5.0. Different group data sets were analysed using either Students’*t*-test or ANOVA. Post-hoc Bonferroni test was used to compare all individual experimental groups amongst each other. *p* value < 0.05 was considered significant. All experiments have been repeated thrice.

## SUPPLEMENTARY MATERIALS



## Abbreviations

miRNA: microRNA
OSCC: Oral squamous cell carcinoma
NC: Negative control
NTC: Non-target control
*Bcl2*: B-cell lymphoma 2
UTR: untranslated region
ECL: enhanced chemo luminescence
TBST: Tris buffer saline-Tween 20
BCA: bichinconic acid
PI: propidium iodide
RE: Restriction Enzyme
FACS: Fluorescence associated cell sorting
