# Correction: Exosome mediated miR-155 delivery confers cisplatin chemoresistance in oral cancer cells via epithelial-mesenchymal transition

**DOI:** 10.18632/oncotarget.28782

**Published:** 2025-11-14

**Authors:** Prathibha Kirave, Piyush Gondaliya, Bhagyashri Kulkarni, Rakesh Rawal, Rachana Garg, Alok Jain, Kiran Kalia

**Affiliations:** ^1^Department of Biotechnology, National Institute of Pharmaceutical Education and Research, Ahmedabad, Gujarat, India; ^2^Department of Life Science, Gujarat University, Ahmedabad, Gujarat, India; ^*^These authors contributed equally to this work and are first authors

**This article has been corrected:** In the FOXO3A blot panel (Figure 2F), the last two lines representing cis Sens +nc and cis Sens +miR-155 mimic are accidental duplicates of the last two lines representing exoRes+nc and exoRes + miR-155 mimic in the FOXO3A blot panel of Figure 4C. The actin band panel in Figure 5D accompanying the β-catenin is an accidental duplicate of the actin band panel of Figure 5B. The corrected Figures 2F and 5D, obtained using the original data, are shown below. The authors declare that these corrections do not change the results or conclusions of this paper.

Original article: Oncotarget. 2020; 11:1157–1171. 1157-1171. https://doi.org/10.18632/oncotarget.27531

**Figure 2:**
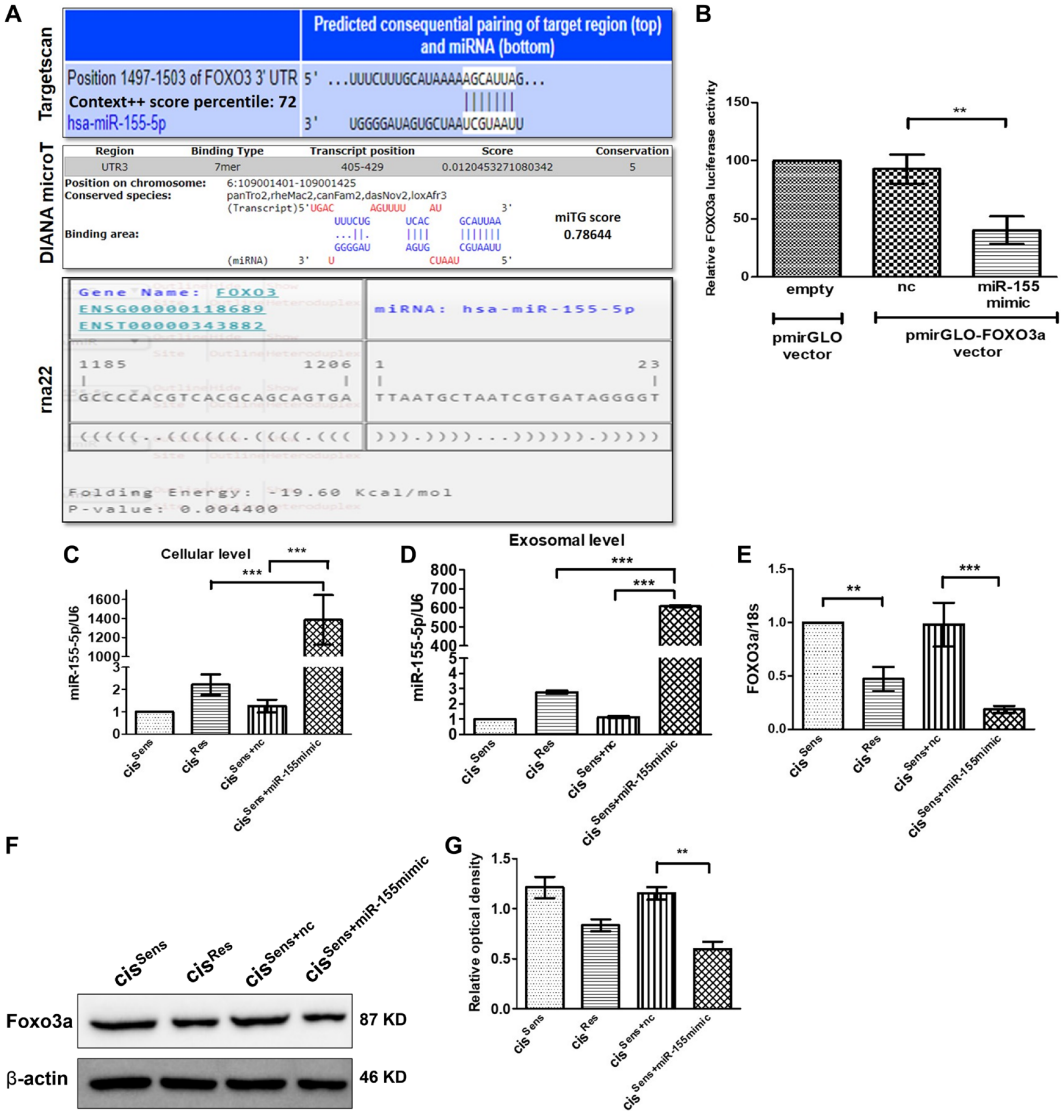
(**A**) Binding position prediction of miR-155 with FOXO3 using TargetScan, DIANA microT-CDS and rna22 web-based tools. (**B**) FOXO3a luciferase activity in cis^Sens^ cells co-transfected with either NTC or miR-155 mimics and the cloned p-mirGLO-FOXO3a dual luciferase vector. Data are expressed as the mean +/− SD. ^**^ *p* < 0.01, significant difference vs. NTC group (*n* = 3). Two independent experiments gave similar results. Following transfection of miR-155 mimics in cis^Sens^ cells, miR-155 expression was validated by q-PCR at both the (**C**) cellular and (**D**) exosomal level. FOXO3a expression was measured by (**E**) q-PCR and (**F**) Western Blot. (**G**) Densitometry analysis of FOXO3a western blot normalized to β-actin as the loading control. Data are expressed as the mean +/− SD. ^*^ *p* < 0.05 and ^**^ *p* < 0.01. (*n* = 3). Two independent experiments gave similar results.

**Figure 5:**
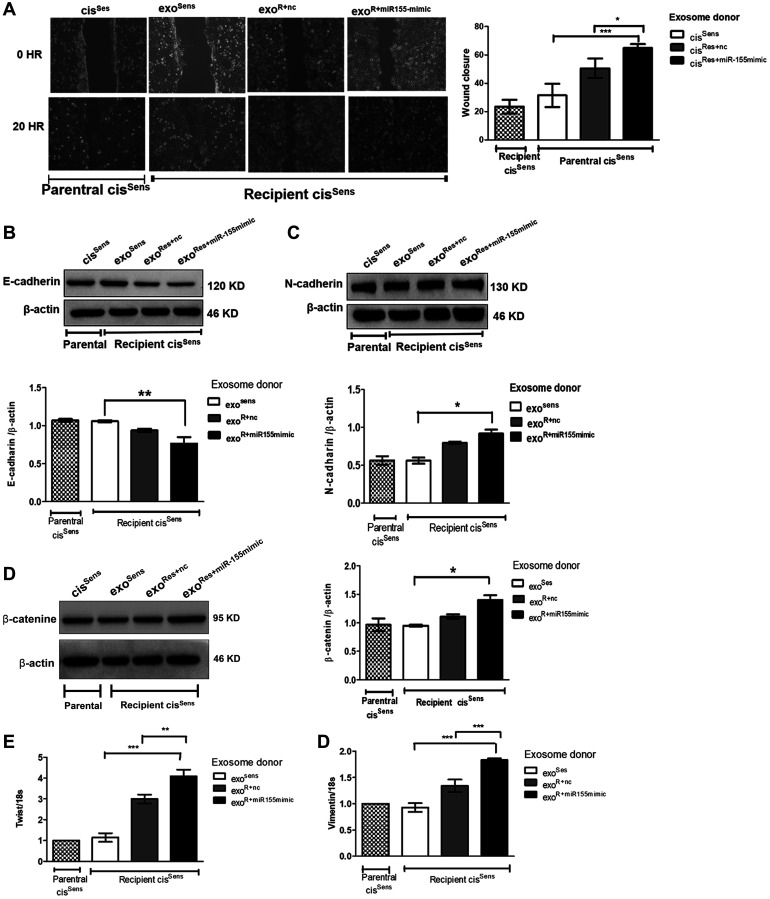
Exosomes were isolated from cis^Sens^ and cis^Res^ cells transfected with either *NTC* or miR-155 mimics, and were used to treat naïve cis^Sens^ cells. cis^Sens^ cells were employed as controls and for comparison. (**A**) Effects of cisplatin treatment was analyzed on the migration of cis^Sens^ oral cancer cells receiving exosomes from cis ^Res+miR-155 mimic^ cells by wound assay. *Left panel*, Representative images of wound closure taken at 0 and 24 h after the scratch was made and cisplatin treatment initiated. *Right panel*, quantification of wound closure as analyzed using Image J. Western Blot expression of various EMT associated markers was measured. Densitometry analysis was carried out with β-actin as loading control. The protein markers included: (**B**) E-cadherin, (**C**) N-cadherin, and (**D**) β-catenin, (**E**) Twist and (**F**) Vimentin expressions were quantified by q-PCR and normalized with respect to 18S as the housekeeping gene. Data are expressed as mean ± SD. ^*^ *p* < 0.05 and ^**^ *p* < 0.01, ^***^ *p* < 0.001 (*n* = 3). Two independent experiments gave similar results.

