# Supplementary material for: Exosome mediated miR-155 delivery confers cisplatin chemoresistance in oral cancer cells via epithelial-mesenchymal transition
Source: Oncotarget. 2020 Mar 31;11(13):1157–71. doi: 10.18632/oncotarget.27531 (PMC7138164; doi:10.18632/oncotarget.27531)
Supplement: Supplementary file 1 [file oncotarget-11-1157-s001.pdf]

Exosome mediated miR-155 delivery confers cisplatin chemoresistance in oral cancer cells via epithelial-mesenchymal transition

SUPPLEMENTARY MATERIALS

Supplementary Table 1: Predicted gene targets of miR-155-5p from various web based tools

Gene Target	Targetscan (Context++ score percentile)	DIANA micro-T (miTG score)	RNA22 (Folding energy) (kcal/mol)
MBTD1	96	0.9991	-
LRP1B	69	0.9990	-
ARID2	98	0.9985	-
FOS	99	0.9978	-
RBAK	91	0.9965	-
STXBP5L	82	0.9964	-
WEE1	99	0.9964	-
RELA	90	0.9952	-
ACTA1	99	0.9602	-
ARID2	98	0.9985	-
H3F3A	99	0.7733	-
TM65F1	99	0.9546	-
FBXO33	99	0.9971	-
DYNC1/1	99	0.9924	-12.90
CARNSP1	99	0.9914	-13.20
TCF7L2	97	0.8689	-
**FOXO3**	**72**	**0.7864**	**-19.60**
FBXO33	99	-	-12.90
FOS	-	0.9978	-14.40
STXBP5L	-	0.9964	-15.20

Symbol – indicate no folding energy was found between miRNA and target sequence.

Supplementary Table 2: Primer sequence of targets

Gene	Forward primer	Reverse primer
FOXO-3a	GAATGTTGTTGGTTTGAACG	ATTTGGCAAAGGGTTTTCTC
Vimentin	GGAAACTAATCTGGATTCACTC	CATCTCTAGTTTCAACCGTC
Twist	CTAGATGTCATTGTTTCCAGAG	CCCTGTTTCTTTGAATTTGG
18s	ATCGGGGATTGCAATTATTC	CTCACTAAACCATCCAATCG

Supplementary Table 3: miR-155 targets and their respective 3′UTR regions

Targets	3′UTR Oligo
**FOXO3A-F**	5′-AAACTAGCGGCCGCAAACTAGCGGCCGCTTTCTTTGCATAAAAAGCATTAGTT -′3
**FOXO3A-R**	3′-TTTGATCGCCGGCGCTAGACTAATGCTTTTTATGCAAAGAAAGCGGCCGCTAGTTTAGATC-′5
